# Electrophysiological evidence for differences between fusion and combination illusions in audiovisual speech perception

**DOI:** 10.1111/ejn.13734

**Published:** 2017-11-06

**Authors:** Martijn Baart, Alma Lindborg, Tobias S. Andersen

**Affiliations:** ^1^ Department of Cognitive Neuropsychology Tilburg University Warandelaan 2 Tilburg 5000 LE The Netherlands; ^2^ BCBL. Basque Center on Cognition, Brain and Language Donostia Spain; ^3^ Section for Cognitive Systems DTU Compute Technical University of Denmark Lyngby Denmark

**Keywords:** audiovisual speech integration, ERPs, P2 suppression, phonetic audiovisual (in)congruency

## Abstract

Incongruent audiovisual speech stimuli can lead to perceptual illusions such as fusions or combinations. Here, we investigated the underlying audiovisual integration process by measuring ERPs. We observed that visual speech‐induced suppression of P2 amplitude (which is generally taken as a measure of audiovisual integration) for fusions was similar to suppression obtained with fully congruent stimuli, whereas P2 suppression for combinations was larger. We argue that these effects arise because the phonetic incongruency is solved differently for both types of stimuli.

## Introduction

When a speech sound (A, for auditory speech) is accompanied by the speaker's articulatory gestures (V, for visual speech), the listener's brain integrates the unimodal signals. Audiovisual (AV) speech integration can lead to percepts that correspond to the phonetic identity of the visual (or auditory) component (e.g. Tuomainen *et al*., 2005; Saint‐Amour *et al*., 2007; Alsius *et al*., 2014), but can also lead to percepts that are different from either A or V. This is evident from a highly influential paper by McGurk & MacDonald (1976) who showed that seeing ‘g’ while the actual speech sound is a ‘b’ (i.e. A_b_V_g_) may yield illusory ‘d’ percepts. This effect is usually referred to as a McGurk fusion (e.g. Green *et al*., 1991; Sekiyama & Tohkura, 1991; van Wassenhove *et al*., 2005; Schwartz, 2010; van Wassenhove, 2013; Tiippana, 2014), as the brain solves the phonetic AV conflict by fusing the place of articulation cues. Such fusions do not always occur; changing the modality of the conflicting consonants can produce a combination percept in which both A and V are represented (i.e. A_g_V_b_ is perceived as ‘bg’ or ‘gb’, e.g. MacDonald & McGurk, 1978).

Colin *et al*. (2002) showed that fusions tend to occur more often with voiced consonants (e.g. ‘b’, ‘g’) whereas combinations are more prominent with voiceless ones (e.g. ‘p’, ‘k’). Moreover, fusions show a left hemifield advantage (when V is presented in the left hemifield) and combinations a right hemifield one (Diesch, 1995). It thus appears that fusion and combination percepts may not necessarily be driven by the exact same processes. Here, we explored whether the electrophysiological correlates of AV integration are different for McGurk fusion and combination stimuli.

Past work has demonstrated that effects of AV speech integration are characterized by V‐induced speeding up and suppression of the auditory‐evoked N1 and P2 peaks (e.g. Klucharev *et al*., 2003; van Wassenhove *et al*., 2005; see Baart, 2016 for a meta‐analysis). Although phonetic AV integration is reflected at the P2 (Baart *et al*., 2014), the complete process requires a subsequent feedback loop that involves STS (Arnal *et al*., 2009). Therefore, we hypothesized that differences in AV integration patterns between McGurk fusions and combinations at/after the P2 could hint at differences related to congruency processing. To investigate this, we compared V‐induced electrophysiological effects for McGurk fusions and combinations with the effects obtained with AV congruent stimuli.

## Materials and methods

### Participants

Thirty eight right‐handed native speakers of Spanish with (corrected to) normal vision and no known hearing or neurological impairments participated in return for a 10€/h payment. All participants provided written informed consent prior to testing. The experiment was conducted in accordance with the Declaration of Helsinki. Six participants were excluded from analyses (four had substantial artefacts in the EEG, one mixed‐up response categories, and one was removed due to software failure). Mean age in the final sample of 32 participants (19 females) was 23.5 years (SD = 0.51).

### Stimuli

A male speaker (MB) was recorded with a digital video camera (videos were framed as headshots) and its internal microphone (Canon Legria HF G10, 25 frames/s) while pronouncing /bi/ and /gi/. With FFmpeg, AV /bi/ and /gi/ video segments were extracted from the recordings, and sounds were extracted from the segments (and equated in maximum intensity). The first three and final two frames of the videos were faded in/out, and the videos were saved as bitmaps strings (30 bitmaps per video). AV stimulus presentations consisted of auditory /bi/ and /gi/ and a simultaneously presented /bi/ or /gi/ bitmap string (40 ms/bitmap, 520 ms of anticipatory motion before sound onset), resulting in two AV congruent stimuli (A_b_V_b_, A_g_V_g_), one fusion stimulus (A_b_V_g_) and one combination stimulus (A_g_V_b_). For V‐only presentations (V_b_, V_g_), the /bi/ and /gi/ bitmaps were delivered in silence, and for auditory‐only presentations (A_b_, A_g_), the bitmap string consisted of black images.

### Procedure

Participants sat in front of a 17‐in. CRT monitor (100 Hz vertical refresh) in a dimly lit booth. Speech sounds were delivered via two regular computer speakers (placed on both sides of the monitor) at an intensity of ~67 dB(A). Videos were 20.6 (W) × 22.5 (H) cm in size. In total, 640 trials were presented in random order. Half of the trials were unimodal (A_b_, A_g_, V_b_, V_g_), and half were bimodal (A_b_V_b_, A_g_V_g_, A_b_V_g_, A_g_V_b_). Each stimulus was presented 80 times. During a trial, a 1200‐ms black screen was followed by a white fixation cross (400 ms) and a period of silence that jittered between 1000 and 1400 ms. Next, the stimulus was presented, which was followed by a response screen that appeared 1000 ms after the last video frame had disappeared. On the response screen, four response categories were presented horizontally in print (‘b’, ‘g’, ‘d’, ‘bg/gb’), and participants indicated which alternative corresponded to their percept. Responses were collected with four fingers of the right hand via the F5 through F8 keys on a regular keyboard (inverted by 180°), and each response category was randomly assigned to a finger for each participant. As soon as a response was collected, the next trial began. There were five ~12‐min. blocks with self‐paced breaks in between. The experiment was preceded by a six‐trial practice block that contained two A, two V and two congruent AV trials.

### EEG recording

The electroencephalogram (EEG) was recorded at a 500 Hz sampling rate using a 32‐channel BrainAmp system (Brain Products GmbH) and 28 Ag/AgCl electrodes that were placed in an EasyCap recording cap. Electrode locations corresponded to a subset of the international 10‐10 placement system and included Fp1, Fp2, F7, F3, Fz, F4, F8, FC5, FC1, FC2, FC6, T7, C3, Cz, C4, T8, CP5, CP1, CP2, CP6, P7, P3, Pz, P4, P8, O1, O2 and FCz (ground). Four electrodes (two on the orbital ridge above and below the right eye and two next to the lateral canthi of both eyes) recorded the vertical and horizontal electro‐oculogram (EOG). Two additional electrodes were placed on the mastoids, of which the left was used to reference the signal online. After placement of the cap, electrode impedance was adjusted to < 5 kΩ (scalp electrodes) and < 10 kΩ (EOG electrodes).

### Pre‐processing of event‐related potentials (ERPs)

Using Brain Vision Analyzer 2.0, the signal was re‐referenced offline to an average of the two mastoid electrodes and high‐pass filtered (0.1 Hz 24 dB/ octave). Next, coarse non‐ocular artefacts (EMG bursts or glitches, defined as amplitude changes > 70 μV/ms) were identified, and the data were decomposed into 32 independent components (restricted infomax). Components that captured EOG activity (2.6 on average, identified through visual inspection) and ECG activity (present in 10 participants, identified at the right mastoid) were removed. The data were low‐pass filtered (30 Hz 24 dB/ octave) and segmented into 1720‐ms epochs. The V as well as the AV epochs contained 200 ms before onset of the video. Auditory onset lagged video onset by 520 ms. Accordingly, the A (and AV) epochs contained 720 ms before sound onset.

Epochs that contained additional artefacts (amplitude changes > 30 μV/ms, and amplitudes </> −100/100 μV, or < 0.5 μV/200 ms) were removed. Four participants with high artefact rates (> 47% per condition) were excluded from analyses. For the remaining participants (*N* = 32), mean artefact rate was < 11% per condition. The data were baseline corrected using the 200‐ms pre‐video time window, averaged per condition and exported for statistical analyses.

## Results and statistical analyses

### Behavioural responses

**Figure 1:**
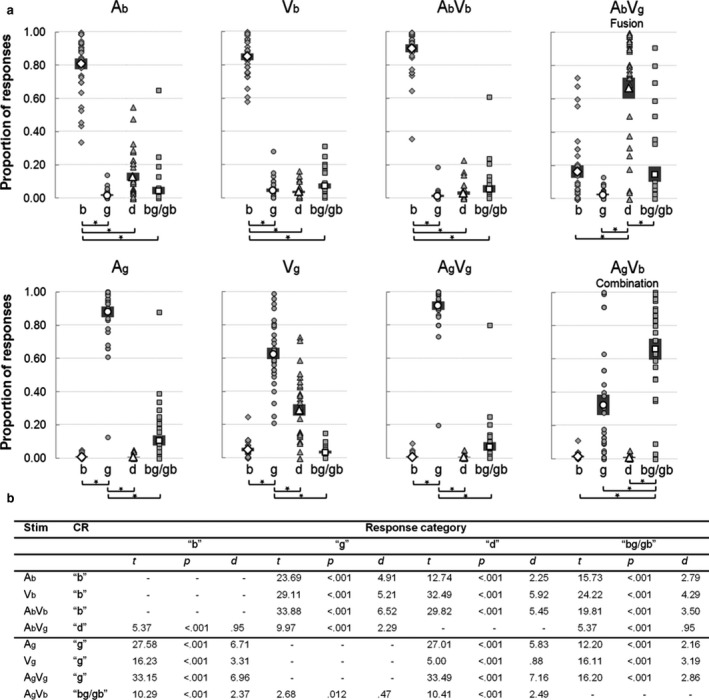
Proportions of ‘b’, ‘g’, ‘d’ and ‘bg/gb’ responses per stimulus. Panel a depicts individual data (grey), averages (white) and standard errors of the mean (shaded areas). Significance of the pairwise comparisons for ‘correct’ vs. ‘incorrect’ responses per stimulus is indicated below the plots. Panel b shows the corresponding test statistics, *P*‐values and effect sizes (all significant after FDR correction). ‘Stim’ indicates stimulus type, and ‘CR’ indicates the correct response to a stimulus.

We computed the averaged proportions of ‘b’, ‘g’, ‘d’ and ‘bg/gb’ responses per stimulus and submitted these data to an 8 (Stimulus; Ab, Ag, Vb, Vg, AbVb, AgVg, AbVg, AgVb) × 4 (Response category; ‘b’, ‘g’, ‘d’, ‘bg/gb’) repeated‐measures anova. The anova revealed an interaction effect, *F*_21,651_ = 156.33, *P *< 0.001, $\eta_{P}^{2}$ = 0.835, indicating that the stimuli were perceived differently, and as intended (see Fig. 1). This was confirmed in eight FDR‐corrected pairwise comparisons that tested the proportion of ‘correct’ responses (i.e. ‘b’ for A_b_, V_b_, A_b_V_b_, ‘g’ for A_g_, V_g_, A_g_V_g_, ‘d’ for A_b_V_g_ and ‘bg/gb’ for A_g_V_b_) against the sum of all other proportions for each stimulus, *t*s(31) > 2.53, *P*s < 0.017, *d*s in between 0.447 and 3.06. Figure 1 additionally displays the 24 comparisons between ‘correct’ responses and all individual response categories.

To compare the strength of the fusion and combination illusions, we also tested the proportions of ‘d’ responses on fusion stimuli against the proportion of ‘bg/gb’ responses on combination stimuli, but this difference was not significant, *t *<* *1.

### ERP data

**Figure 2:**
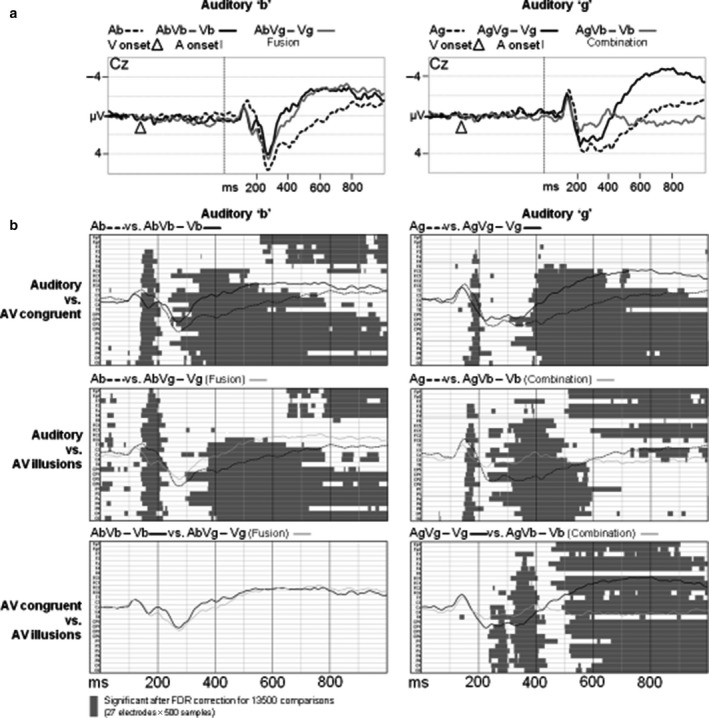
Auditory grand averages, AV – V difference waves and statistical comparisons. Panel a shows the waveforms at Cz for stimuli with auditory ‘b’ (left column) and auditory ‘g’ (right column). In panel b, time zero corresponds to sound onset, and grey horizontal bars represent significant differences between conditions. For each pairwise comparison, the ERPs from electrode Cz are overlaid.

Following an additive model, AV integration effects can be captured by comparing A‐only ERPs with AV – V difference waves (e.g. Besle *et al*., 2004; Stekelenburg & Vroomen, 2007; Giard & Besle, 2010; Alsius *et al*., 2014; Baart *et al*., 2014). Figure 2a displays the A‐only grand averages and the AV – V difference waves at electrode Cz for the conditions with auditory ‘b’ (A_b_, A_b_V_b_ – V_b_ and A_b_V_g_ – V_g_; left panel) and auditory ‘g’ (A_g_, A_g_V_g_ – V_g_ and A_g_V_b_ – V_b_; right panel).

As indicated in Fig. 2a, the averaged P2 peaks for A_g_, A_g_V_g_, and A_g_V_b_ – V_b_ are not as well‐defined as for A_b_, A_b_V_b_, and A_b_V_g_ – V_g_. Because individual peaks in those conditions could not always be determined, our analyses contained two steps. First, we cast a wide temporal net around the effects of interest by computing the average amplitude at electrode Cz in relatively large time windows around the N1 (100–200 ms) and P2 (200‐300 ms), and we analysed those amplitudes in repeated‐measures anovas. The second step comprised of a more detailed analyses between conditions using FDR‐corrected pairwise *t*‐tests that included all electrodes (see Fig. 2b).

For the N1, a 3 (Stimulus type; A, AV congruent [i.e. A_b_V_b_ – V_b_ and A_g_V_g_ – V_g_], AV incongruent [i.e. A_b_V_g_ – V_g_ and A_g_V_b_ – V_b_]) × 2 (Auditory component, /b/ or /g/) repeated‐measures anova showed a main effect of Stimulus type, *F*_2,62_ = 6.15, *P *=* *0.004, $\eta_{P}^{2}$ = 0.166, because V had suppressed the N1 for both congruent and incongruent stimuli, *t*s(31) > 2.39, *P*s < 0.024, *d*s > 0.428. There was also a main effect of Auditory component, *F*_1,31_ = 16.05, *P *<* *0.001, $\eta_{P}^{2}$ = 0.341, as overall N1 amplitude was larger for auditory ‘g’ than ‘b’ stimuli. There was no interaction between the two factors, *F *<* *1. This was confirmed in a 2 (Stimulus type; AV congruent, AV incongruent) × 2 (Auditory component, /b/ or /g/) anova without the A‐only data, which also revealed no interaction, *F *<* *1.

For the P2, the 3 × 2 anova also yielded a main effect of Stimulus type, *F*_2,62_ = 6.39, *P *=* *0.003, $\eta_{P}^{2}$ = 0.171, as V had suppressed the P2 for congruent and incongruent stimuli, *t*s(31) > 2.63, *P*s < 0.012, *d*s > 0.465. There was a main effect of Auditory component, *F*_1,31_ = 30.90, *P *<* *0.001, $\eta_{P}^{2}$ = 0.499, as the overall P2 amplitude was larger for auditory ‘b’ stimuli than for auditory ‘g’ stimuli. The interaction was significant, *F*_2,62_ = 5.57, *P *=* *0.006, $\eta_{P}^{2}$ = 0.152, and was also observed when the auditory‐only stimuli were omitted from the anova,* F*_1,31_ = 6.45, *P *=* *0.016, $\eta_{P}^{2}$ = 0.172, because for A_b_, P2 amplitude was alike for congruent stimuli and fusion stimuli, *t*(31) = 1.63, *P *=* *0.113, *d *=* *0.289, whereas for A_g_ P2 suppression was larger for combinations than for congruent stimuli, *t*(31) = 2.71, *P *=* *0.011, *d *=* *0.490.

The results of the FDR‐corrected pairwise *t*‐tests are displayed in Fig. 2b and confirm that V had indeed suppressed (and possibly sped up the N1 and) P2. Most importantly, P2 suppression was larger for McGurk combinations (A_g_V_b_ – V_b_) than for congruent A_g_V_g_ – V_g_, whereas there were no significant differences between fusions (A_b_V_g_ – V_g_) and congruent A_b_V_b_ – V_b_. When averaging amplitude at Cz in a 190‐ to 200‐ms and 360‐ to 440‐ms window however, the differences between A_b_V_b_ – V_b_ and fusions were significant, *t*s(31) > 2.21, *P*s < 0.035, *d*s > 0.391, but these differences did not correspond to the ERP peaks under investigation, and lost significance in the FDR correction.

## Discussion

We sought to determine whether the electrophysiological correlates of AV integration at the N1/P2 are different for McGurk fusions and combinations. V‐induced suppression of the N1/P2 is generally interpreted as an effect of AV integration, and from that perspective, it is evident that A and V are integrated in both types of McGurk stimuli. There was, however, one important difference between fusions and combinations.

For fusions, P2 suppression was equal to the suppression effect for congruent A_b_V_b_. This is in line with Fig. 2 in van Wassenhove *et al*. (2005) where the fusion AV P2 amplitude (A_p_V_k_, fusion percept = ‘t’) is more similar to the amplitude of congruent stimuli with the same auditory component (A_p_V_p_), than to the amplitude of the AV congruent stimulus with same auditory component as the fusion (A_t_V_t_). For combinations however, we observed that P2 suppression was significantly larger than the effect observed with congruent A_g_V_g_.

Interestingly, V‐induced suppression of the auditory P2 is larger for AV incongruent than congruent speech (such as when auditory ‘fu’ is combined with lip‐read ‘bi’, see Stekelenburg & Vroomen, 2007). The current findings therefore suggest that the AV incongruency in combination stimuli has a different impact than the incongruency in fusion stimuli: relatively early processing (measured at the P2) of combination stimuli resembles the pattern observed with fully phonetically AV incongruent material (Stekelenburg & Vroomen, 2007), whereas the P2 suppression for fusions resembles the pattern of AV congruent speech. It may thus be that the differences between fusions and combinations reflect differences in processing of AV congruency, which is supported by the clear differences between the combination difference wave and all others *after* the P2 (in line with Arnal *et al*., 2009, who argued that processing of AV congruency requires multiple feedback loops).

However, it is possible that listeners did not notice the AV incongruency in combination stimuli (this was not measured), and the differences between fusions and combinations may therefore be explained by other stimulus features. For example, in consonant–vowel stimuli, the (latency) and amplitude of the N1/P2 can reflect stimulus differences in voice‐onset time (e.g. Tremblay *et al*., 2003; Digeser *et al*., 2009), amplitude rise time and rate of formant transition (Carpenter & Shahin, 2013). If such basic acoustic features modulate the P2, it is possible that the difference between the McGurk combinations and fusions is related to the fact that in combinations, *two* consonants instead of one are perceived, despite that physically, all our AV stimuli contained only one auditory consonant. Related to this, fusion likely occurs in AV congruent stimuli as well as in the McGurk fusion stimulus, and the combination stimulus is thus fundamentally different that all other stimuli.

However, the interpretations outlined above are speculative at this point as they require future work to determine how the number of consonants modulates the ERPs (e.g. by including genuine ‘bg’ or ‘gb’ AV congruent speech), and the degree to which combination ERPs resemble those obtained with AV incongruent stimuli in which the phonetic incongruency is impossible to overcome (e.g. ‘bi’ vs. ‘fu’).

To conclude, we observed that the ERP pattern of AV integration for McGurk fusions clearly differs from combinations. It is unlikely that these differences stem from actual differences in the underlying integration process. Instead, the inherent differences between fusion and combination stimuli differentially constrain the perceptual system when it is trying to solve the AV incongruency.

## Conflict of interest

The authors declare no conflict of interests.

## Author contribution

The study was designed by MB, AL and TSA. MB created the stimuli. AL programmed the experiment. MB oversaw data collection and analysed the data. MB, AL and TSA wrote the manuscript.

## Data accessibility

Anonymized data, stimuli and details about pre‐processing/analyses will be made available to colleagues if requested.

**Abbreviations**
A: auditory
AV: audiovisual
EEG: electroencephalogram
EMG: electromyogram
EOG: electro‐oculogram
ERP: event‐related potential
V: visual lip‐read information

## References

[ejn13734-bib-0001] Alsius, A. , Möttönen, R. , Sams, M.E. , Soto‐Faraco, S. & Tiippana, K. (2014) Effect of attentional load on audiovisual speech perception: evidence from ERPs. Front. Psychol., 5, 727. 25076922 10.3389/fpsyg.2014.00727 PMC4097954

[ejn13734-bib-0002] Arnal, L.H. , Morillon, B. , Kell, C.A. & Giraud, A.L. (2009) Dual neural routing of visual facilitation in speech processing. J. Neurosci., 29, 13445–13453. 19864557 10.1523/JNEUROSCI.3194-09.2009 PMC6665008

[ejn13734-bib-0003] Baart, M. (2016) Quantifying lip‐read‐induced suppression and facilitation of the auditory N1 and P2 reveals peak enhancements and delays. Psychophysiology, 53, 1295–1306. 27295181 10.1111/psyp.12683

[ejn13734-bib-0004] Baart, M. , Stekelenburg, J.J. & Vroomen, J. (2014) Electrophysiological evidence for speech‐specific audiovisual integration. Neuropsychologia, 53, 115–121. 24291340 10.1016/j.neuropsychologia.2013.11.011

[ejn13734-bib-0005] Besle, J. , Fort, A. & Giard, M.H. (2004) Interest and validity of the additive model in electrophysiological studies of multisensory interactions. Cogn. Process., 5, 189–192.

[ejn13734-bib-0006] Carpenter, A.L. & Shahin, A.J. (2013) Development of the N1–P2 auditory evoked response to amplitude rise time and rate of formant transition of speech sounds. Neurosci. Lett., 544, 56–61. 23570734 10.1016/j.neulet.2013.03.041 PMC3756151

[ejn13734-bib-0007] Colin, C. , Radeau, M. , Deltenre, P. , Demolin, D. & Soquet, A. (2002) The role of sound intensity and stop‐consonant voicing on McGurk fusions and combinations. Eur. J. Cogn. Psychol., 14, 475–491.

[ejn13734-bib-0008] Diesch, E. (1995) Left and right hemifield advantages of fusions and combinations in audiovisual speech perception. Q. J. Exp. Psychol., 48, 320–333. 10.1080/14640749508401393 7610270

[ejn13734-bib-0009] Digeser, F.M. , Wohlberedt, T. & Hoppe, U. (2009) Contribution of spectrotemporal features on auditory event‐related potentials elicited by consonant‐vowel syllables. Ear Hearing, 30, 704–712. 19672195 10.1097/AUD.0b013e3181b1d42d

[ejn13734-bib-0010] Giard, M.H. & Besle, J. (2010). Methodological considerations: electrophysiology of multisensory interactions in humans In Kaiser J. & Naumer M.J. (Eds), Multisensory Object Perception in the Primate Brain. Springer, New York, pp. 55–70.

[ejn13734-bib-0011] Green, K.P. , Kuhl, P.K. , Meltzoff, A.N. & Stevens, E.B. (1991) Integrating speech information across talkers, gender, and sensory modality: female faces and male voices in the McGurk effect. Percept. Psychophys., 50, 524–536. 1780200 10.3758/bf03207536

[ejn13734-bib-0012] Klucharev, V. , Möttönen, R. & Sams, M. (2003) Electrophysiological indicators of phonetic and non‐phonetic multisensory interactions during audiovisual speech perception. Cognitive Brain Res., 18, 65–75. 10.1016/j.cogbrainres.2003.09.004 14659498

[ejn13734-bib-0013] MacDonald, J. & McGurk, H. (1978) Visual influences on speech perception processes. Percept. Psychophys., 24, 253–257. 704285 10.3758/bf03206096

[ejn13734-bib-0014] McGurk, H. & MacDonald, J. (1976) Hearing lips and seeing voices. Nature, 264, 746–748. 1012311 10.1038/264746a0

[ejn13734-bib-0015] Saint‐Amour, D. , De Sanctis, P. , Molholm, S. , Ritter, W. & Foxe, J.J. (2007) Seeing voices: high‐density electrical mapping and source‐analysis of the multisensory mismatch negativity evoked during the McGurk illusion. Neuropsychologia, 45, 587–597. 16757004 10.1016/j.neuropsychologia.2006.03.036 PMC1705816

[ejn13734-bib-0016] Schwartz, J.‐L. (2010) A reanalysis of McGurk data suggests that audiovisual fusion in speech perception is subject‐dependent. J. Acoust. Soc. Am., 127, 1584–1594. 20329858 10.1121/1.3293001

[ejn13734-bib-0017] Sekiyama, K. & Tohkura, Y.I. (1991) McGurk effect in non‐English listeners: few visual effects for Japanese subjects hearing Japanese syllables of high auditory intelligibility. J. Acoust. Soc. Am., 90, 1797–1805. 1960275 10.1121/1.401660

[ejn13734-bib-0019] Stekelenburg, J.J. & Vroomen, J. (2007) Neural correlates of multisensory integration of ecologically valid audiovisual events. J. Cognitive Neurosci., 19, 1964–1973. 10.1162/jocn.2007.19.12.1964 17892381

[ejn13734-bib-0020] Tiippana, K. (2014) What is the McGurk effect? Front. Psychol., 5, 725. 25071686 10.3389/fpsyg.2014.00725 PMC4091305

[ejn13734-bib-0021] Tremblay, K.L. , Friesen, L. , Martin, B.A. & Wright, R. (2003) Test‐retest reliability of cortical evoked potentials using naturally produced speech sounds. Ear Hearing, 24, 225–232. 12799544 10.1097/01.AUD.0000069229.84883.03

[ejn13734-bib-0022] Tuomainen, J. , Andersen, T.S. , Tiippana, K. & Sams, M. (2005) Audio–visual speech perception is special. Cognition, 96, B13–B22. 15833302 10.1016/j.cognition.2004.10.004

[ejn13734-bib-0023] van Wassenhove, V. (2013) Speech through ears and eyes: interfacing the senses with the supramodal brain. Front. Psychol., 4, 388. 23874309 10.3389/fpsyg.2013.00388 PMC3709159

[ejn13734-bib-0024] van Wassenhove, V. , Grant, K.W. & Poeppel, D. (2005) Visual speech speeds up the neural processing of auditory speech. Proc. Natl. Acad. Sci. USA, 102, 1181–1186. 15647358 10.1073/pnas.0408949102 PMC545853

