# Supplementary material for: Electrophysiological evidence for differences between fusion and combination illusions in audiovisual speech perception
Source: Eur J Neurosci. 2017 Nov 6;46(10):2578–83. doi: 10.1111/ejn.13734 (PMC5725699; doi:10.1111/ejn.13734)
Supplement: Supplementary file 1 [file EJN-46-2578-s001.pdf]

EJN European Journal of Neuroscience FENS Federation of European Neuroscience Societies

Electrophysiological evidence for differences between fusion and combination illusions in audiovisual speech perception

Martijn Baart, Alma Lindborg and Tobias S. Andersen

Review timeline:

Submission date:	08 May 2017
Editorial Decision:	25 June 2017
Revision received:	30 August 2017
Editorial Decision:	20 September 2017
Revision received:	27 September 2017
Accepted:	27 September 2017

Editor: John Foxe

1st Editorial Decision 25 June 2017

Dear Dr. Baart,

Your manuscript has now been reviewed by three external reviewers as well as by editors. While all of the reviewers see merit in the work, there are several serious issues that need to be clarified/resolved before we can consider your manuscript further for publication in EJN. The most significant issue is raised by Reviewer #3, who questions whether there may have been a calculation error in the derivation of your subtraction waveforms, since these do not accord well with the prior literature and do not appear to be internally consistent. Please check this issue very carefully and provide a clear explanation so that we can be confident that the derivations have been calculated correctly. Please clarify how trials for inclusion were defined in the main conditions, and why the "capture" conditions were not included in your report. Please also provide a more complete discussion of the extant literature. The general sparsity of the introduction and discussion sections was picked up on by all of the reviewers. The introduction does not do a sufficient job of laying out the theoretical framework under which the study was executed, and this is also an issue with the abstract. Please remove references for the abstract This editor was also somewhat surprised that the paper by Saint-Amour and colleagues, Neuropsychologia (2007) did not merit inclusion. We will need a more complete presentation of your statistical analyses, reporting effect sizes, and making clear how multiple comparisons were controlled for. Again, an important issue here is the statistical model -- the use of independent tests is not appropriate and an overall Analysis of Variance needs to be conducted so that conditions and potential interactions can be appropriately compared. Lastly, replace your barcharts with scatterplots or some other more informative hybrid depictions - see http://onlinelibrary.wiley.com/doi/10.1111/ejn.13400/epdf

If you are able to respond fully to the points raised, we would be pleased to receive a revision of your paper within 12 weeks.

Thank you for submitting your work to EJN.

Kind regards,

John Foxe & Paul Bolam
co-Editors in Chief, EJN

Reviews:

Reviewer: 1 (Kaisa Tiippana, University of Helsinki, Finland)

Comments to the Author
The manuscript “Electrophysiological evidence for differences between McGurk fusions and combinations” describes a well-designed study, which is appropriate for EJN. The main finding is that P2 suppression was greater for McGurk combination than fusion stimuli. Overall, the manuscript is clear but two points should be clarified. In addition, I have some, more minor suggestions.

First, it is unclear whether all trials were analyzed, or only those in which the response corresponded to the expected one (e.g. for the fusion stimulus, whether only “d” responses were included). The latter would be recommended if the number of trial allows it. Please clarify.

Second, the discussion should be clarified. Particularly, the authors write already in the abstract: “suppression of P2 amplitude (which is generally taken as a measure of AV integration)”, but going on into the discussion, their point seems to be that stronger P2 suppression is related to weaker integration. This change in the interpretation should be explained better. Also, the relationship between the current findings and previous studies using incongruent or combination and fusion stimuli should be described in more detail (e.g. Klucharev & al., 2003). Is their explanation related to the salience or predictability of visual speech (e.g. Arnal & al., 2009)? Further, the end of the first paragraph on p. 11 is unclear: “audiovisual integration occurs, at least partly, after the P2…”

More minor suggestions:
The final sentence of the abstract could be more specific.

A McGurk stimulus may be heard according to the visual component (e.g. Alsius & al., 2014), which should be mentioned.

p. 6 “participants indicated which alternative corresponded to their auditory percept”. Except in V trials.

p. 8 Were the pair-wise comparisons corrected in the behavioral responses (see p. 9 FDR)?

p. 8 “Following an additive model, AV integration effects can be captured by comparing A-only ERPs with AV-V difference waves”. However, the first author has an excellent paper (Baart, 2016), according to which subtracting V is not needed. Why did the authors choose to use AV-V rather than AV here? I would like to know, but do not ask that this explanation should be included in the manuscript text unless the authors opt to do so.

p. 9-10 The authors may consider adding the significant P2 time windows in the text, and aligning the timelines in Fig. 2 a and b. Also, adding “stimulus” after McGurk fusion/combinations might make the end of the Results clearer.

Reviewer: 2 (Ryan Stevenson, University of Western Ontario, Canada)

Comments to the Author
The manuscript entitled ”Electrophysiological evidence for differences between McGurk fusions and combinations” compares the ERP waveforms in response to fused vs combination perceptions of the McGurk Effect. The methodology appears completely sound, as does eh analysis performed. With that said, the supporting sections, in terms of introduction and interpretation of results needs to be greatly bolstered. Specific comments are below.

Introduction
Recognizing that the analysis focuses on the comparison between fusions and combinations, it seems odd that the cases of visual or auditory capture are not discussed, as they are generally far more common than the combination perception.

The neuronal underpinning of sensory integration in general, and McGurk in specific need to be introduced in the intro. As it is, there is only a single sentence in the last paragraph, but there is an extensive literature. What is the N1-P2 thought to represent? What is the source? What other multisensory ERP effects have been observed with McGurk? What is the hypothesized underlying difference between fusion and combos, etc.

Methods:
Pg 5 – where were the speakers located?

Pg 7 – Why was the re-referencing not done to the global mean?

Results:
Please report effect sizes for all stats – given the large number of follow up t-tests, perhaps a table would allow for the more precise reporting of statistics, with the pertinent summary wording still in the text?

Why was the choice made to compare AV directly to V as opposed to comparing it to (A+V)?

Discussion:

EJN European Journal of Neuroscience FENS Federation of European Neuroscience Societies

One difference between fusion and combinations that is not discussed is that the total number of phonemes perceived is the definition of fusion. Fusion occurs in all congruent presentations and the “fusion”perceptions - except for the combination. It’s not simply that the combination perception also includes an extra phoneme perceived, which is completely accurate, but it’s also that fusion occurs in the congruent trial. The combination trial is fundamentally different from the congruent trials in a way that the fusion trial is not.

Pg 10 – if the p2 is thought to represent feedback from STS, this should be discussed in the introduction.

Pg 11 – “However, participants usually do not notice the AV incongruency in combination stimuli (or in fusion stimuli).” – was this measured?

Pg 11 – define CV – Is this a typo that’s supposed to be “AV” or do you mean “consonant-vowel”?

Pg 12 – The discussion of correlational results is rather lacking. Given the graphs of individual data points, I am convinced of the fusion correlation, but not of the combination correlation (see comment about Fig 3 below). Regardless of how these correlations hold up, there needs to be much more in the way of interpreting them for this presented result to be of meaning for the reader.

Fig 3 – It seem that the correlation with combination responses is driven by a minority of participants (7) that reported this perception at any meaningful level.

Typographic:
Pg 3, “Such fusions do not always occur: changing the modality” – should be a semicolon.
Pg 5 – I think “FFMPEG” is written “FFmpeg”

Reviewer: 3 (Daniel Senkowski, Charité- Universitätsmedizin Berlin, Germany)

Comments to the Author
This manuscript describes an EEG study examining the ERP correlates during fusion and combination trials in the McGurk illusion. The research question is interesting and the work could be, in my opinion of potential interest for publication in EJN. However, I have a few points that should be addressed.

Major points:

1. I was wondering whether something have gone wrong in the analysis of AgVb minus Vb trials. I have seen many AV minus V subtraction approaches in AV speech studies but have, thus far, never observed such a large difference between AV minus V vs. A alone as illustrated in Fig. 2, right panel, light gray trace. The trace for the AgVb minus Vb trials simply does not fit to the other six traces (of Auditory “b” and Auditory “g”, which should actually be labeled “combinations and “fusion”). This could also explain why there is such a highly significant difference between Auditory vs. AV congruent for fusion trials, whereas there is no such difference for combination trials. Overall, these results raise skepticism and I would like to encourage the authors to thoroughly double-check all their analysis scripts.

2. In order to draw the conclusion that there are differences between combination and fusion trials the authors should run an ANOVA showing an interaction (instead of comparing the outcome of two t-tests; see Nieuwenhuis et al. Nat. Neurosci., 2011, 1105-1107).

3. I was wondering whether the correlations would still be significant if the authors would have corrected for multiple testing.

4. Do the authors have an explanation why there was not a McGurk fusion effect in the ERPs (i.e. compared to congruent). Such effects have been consistently reported.

Minor issues:
1. Use the labels “fusion” and “combination” consistently in all figures.

2. The Abbreviations “A” and “V” should be spelled-out at first use in the abstract.

3. P. 6.: “...was randomly assigned to a finger.” Was this done across subjects? If so, this should be stated.

4. P. 7: “The V and AV epochs contained 200 ms...”. It is unclear why the AV epochs were computed twice.

EJN European Journal of Neuroscience FENS Federation of European Neuroscience Societies

Authors' Response 30 August 2017

Reply to Reviewer 1

Major

First, it is unclear whether all trials were analyzed, or only those in which the response corresponded to the expected one (e.g. for the fusion stimulus, whether only "d" responses were included). The latter would be recommended if the number of trial allows it. Please clarify.

Response: We apologize for the confusion and we would like to clarify that we analyzed all data, not just those trials that were perceived 'correctly'. Although our approach is in-line with much of the relevant work in which responses are not taken into account either (e.g., Colin et al., 2002, 2004; Saint-Amour et al., 2007; Stekelenburg & Vroomen, 2007), the reviewer has a point that analyses of 'correct' responses may be ideal if the number of trials allow it.

In addition to this however, ERPs should ideally also represent averages across comparable numbers of trials per condition for each participant (which also includes V-only; see our response to this Reviewer's comment below where we explain why the AV - V subtraction is critical). If we were to include only those participants who had at least 40 'correct' trials in all conditions (which is 50% 'correct' or more, and 40 trials are quite minimal to base an ERP on), we would end-up including only half of our sample (N = 17). As can be seen in the figure below, the critical trends in the grand averages that included all data were quite similar to those that included correct responses-only, but we prefer including all participants for the sake of power.

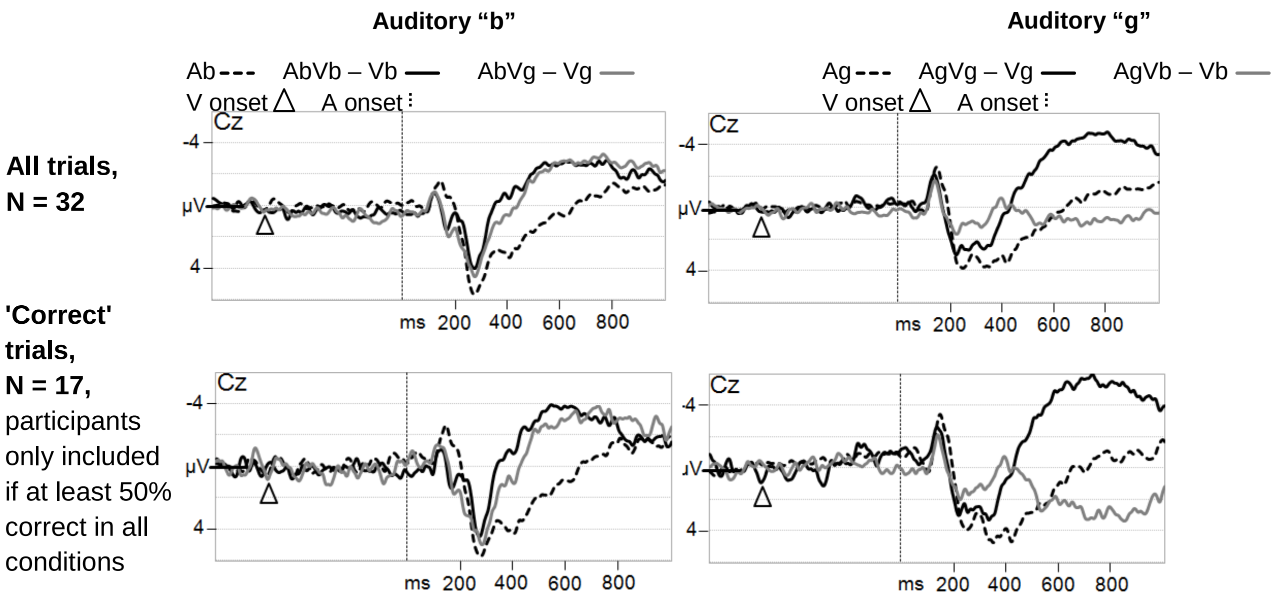

Second, the discussion should be clarified. Particularly, the authors write already in the abstract: “suppression of P2 amplitude (which is generally taken as a measure of AV integration)”, but going on into the discussion, their point seems to be that stronger P2 suppression is related to weaker integration. This change in the interpretation should be explained better. Also, the relationship between the current findings and previous studies using incongruent or combination and fusion stimuli should be described in more detail (e.g. Klucharev & al., 2003). Is their explanation related to the salience or predictability of visual speech (e.g. Arnal & al., 2009)? Further, the end of the first paragraph on p. 11 is unclear: “audiovisual integration occurs, at least partly, after the P2…”

Response: In the abstract, we raise the general point that P2 amplitude suppression is a measure of AV integration in order to justify our approach and analyses. Therefore, we believe this sentence does not need to be changed. We do however, agree with the reviewer that the fact that stronger suppression might be related to weaker integration needs to be clarified. In fact, we cannot really claim that stronger suppression equals weaker integration per se, but only that P2 suppression may reflect differences in the extent to which AV integration on a phonetic level occurs.

Firstly, when there is no phonetic integration (because the listener is unaware of the phonetic content of artificial speech stimuli), there is no P2 suppression (Baart et al., 2014), which indicates that P2 suppression is related to phonetic integration. Secondly, Stekelenburg and Vroomen (2007) showed that incongruent stimuli (/bi/ and /fu/) yielded stronger suppression of the P2 than congruent stimuli. However, if stimuli consisted of vowels only, AV congruent and incongruent materials yield quite similar P2 peaks (Klucharev et al., 2003, Figure 2). In our stimuli, the incongruence was in the initial consonant, and if we then compare our data with the pattern observed by Stekelenburg and Vroomen (2007), it is possible that the larger P2 suppression for combinations than for all other stimuli is related to the fact that phonetic incongruency in the consonants was larger for combinations than for fusions. This seems supported by the fact that Arnal et al. (2009) argue that processing of AV incongruency takes time and requires multiple feedback loops involving STS, which indeed aligns with our data where we see that the combination difference wave is very different from all the others, in the time-frame *after* the P2.

However, the fact remains that we simply cannot be certain whether 'congruency processing' is indeed at the foundation of the differences we observed, as there are multiple reasons why P2 suppression could be different for combinations than for all other stimuli, as we explain in the discussion. We are quite confident though, that temporal predictability (i.e., the fact that the visual signal leads the auditory onset) cannot explain the patterns in the data for two reasons: (1) visual lead (anticipatory motion) was the same across all stimuli, and (2) temporal properties of the stimuli modulate the N1, but not the P2 (e.g., Baart et al., 2014; Stekelenburg & Vroomen, 2007; Vroomen and Stekelenburg, 2010). This however, does not exclude predictability or saliency on a *phonetic* level as a potential explanation, but again, we cannot be sure given the data we have in hand.

Minor

The final sentence of the abstract could be more specific.

Response: We have added the different possible explanations between parentheses.

EJN European Journal of Neuroscience FENS Federation of European Neuroscience Societies

A McGurk stimulus may be heard according to the visual component (e.g. Alsius & al., 2014), which should be mentioned.

Response: We assume the reviewer refers to the work by in which incongruent stimuli comprised a /mi/-/ni/ contrast. Such materials (i.e., stimuli in which the AV phonetic contrast is different from the specific labial/velar conflicts needed to produce McGurk fusions/combinations) can indeed lead to visual capture, and we now include a general statement about unimodal capture on page 3.

p. 6 "participants indicated which alternative corresponded to their auditory percept". Except in V trials.

Response: We thank the Reviewer for identifying this mistake, which we corrected by deleting "auditory".

p. 8 Were the pair-wise comparisons corrected in the behavioral responses (see p. 9 FDR)?

Response: We now clarify that all pair-wise comparisons are indeed FDR corrected. The changes are reflected on page 8 and in Figure 1b.

"Following an additive model, AV integration effects can be captured by comparing A-only ERPs with AV-V difference waves". However, the first author has an excellent paper (Baart, 2016), according to which subtracting V is not needed. Why did the authors choose to use AV-V rather than AV here? I would like to know, but do not ask that this explanation should be included in the manuscript text unless the authors opt to do so.

Response: In a recent meta-analyses, the first author indeed found that A vs. AV and A vs. AV – V comparisons yielded similar effects. However, the data included in that paper were always obtained from AV congruent stimuli. In contrast, McGurk stimuli are incongruent by definition. As a result, any differences that arise from direct comparisons between A_bV_b and A_bV_g, or between A_gV_g and A_gV_b can potentially reflect processing differences between the visual components of the stimuli. Therefore, we subtracted out the visual activity in the AV – V difference waves.

p. 9-10 The authors may consider adding the significant P2 time windows in the text, and aligning the timelines in Fig. 2 a and b. Also, adding "stimulus" after McGurk fusion/combinations might make the end of the Results clearer.

Response: Aligning the time-lines as the Reviewer suggests could implies that the significance plots in Figure 2b become too small to read. However, we added the ERPs for each comparison to Figure 2b, which should clarify the time-windows where effects were significant. In the main text, we now include ANOVAs on fixed time-windows for the N1 (100-200 ms) and P2 (200-300 ms), which produce the same pattern of significance as visible in Figure 2b. We therefore did not add more details in the text as we believe the ANOVAs and the information in Figure 2b provide sufficient information to the reader to understand the effects of interest without ambiguities. We also believe our new results section is clearer than before.

EJN European Journal of Neuroscience FENS Federation of European Neuroscience Societies

Reply to Reviewer 2

Introduction

Recognizing that the analysis focuses on the comparison between fusions and combinations, it seems odd that the cases of visual or auditory capture are not discussed, as they are generally far more common than the combination perception.

Response: In our data, it is clear that, on average, fusions/combinations occurred much more often than capture, and occurred with a frequency that resembles the original report by McGurk and MacDonald (1976). However, the Reviewer is right that auditory or visual capture can occur more often than fusions or combinations (depending on the stimuli that are used), and we therefore discuss the issue briefly on page 3.

The neuronal underpinning of sensory integration in general, and McGurk in specific need to be introduced in the intro. As it is, there is only a single sentence in the last paragraph, but there is an extensive literature. What is the N1-P2 thought to represent? What is the source? What other multisensory ERP effects have been observed with McGurk? What is the hypothesized underlying difference between fusion and combos, etc.

Response: We would like to clarify that the word-limit of the manuscript format does not allow us to describe the full background on the N1 and P2. However, we now do include the congruence processing differences hypothesis in the introduction (page 4), which is based on the fact that AV congruency is processed at, and after, the P2 peak (but not at the N1). The source of the N1/P2 is well-described in the literature, and we believe that the most important fact for the current manuscript is that these are auditory peaks (originating in auditory areas), which are modulated by visual speech. Since EJN's policy is to make the reviewer comments and our responses to those available (if the manuscript gets accepted), we would like to refer to our response to comment #2 by Reviewer 1, where we expand more on the relevant literature and our line of thought.

Methods:

Pg 5 – where were the speakers located?

Response: The speakers were located left and right of the monitor, which is now clarified on page 5.

Pg 7 – Why was the re-referencing not done to the global mean?

Response: When re-referencing to the global mean, the head is considered a concentric sphere with homogeneous conductive properties, and the electrical field generated by the assembled (horizontal and vertical) dipoles would therefore approach zero. The more electrodes are included, the better this underlying principle is met. Since we have a relatively small number of electrodes, we opted for re-referencing against the average of the two mastoids (see also Baart & Samuel, 2015, among many others, where a similar re-referencing procedure is used with the identical EEG set-up).

EJN European Journal of Neuroscience FENS Federation of European Neuroscience Societies

Results:

Please report effect sizes for all stats – given the large number of follow up t-tests, perhaps a table would allow for the more precise reporting of statistics, with the pertinent summary wording still in the text?

Response: The result sections have been changed and effect-sizes are added consistently. The table with the relevant information (e.g., test statistics, *p*-values and effect sizes) is now included in Figure 1.

Why was the choice made to compare AV directly to V as opposed to comparing it to (A+V)?

Response: We never compared to AV directly to V. In the behavioral data, we assessed the proportion of responses per stimulus type, separately for all stimuli, and in the EEG data, we compared A ERPs to the AV – V difference wave. If one assumes that (1) A amplitude > than AV amplitude (which is the basic notion behind N1/P2 peak suppression), and (2) A ≠ A + V (the rationale behind the additive model), comparing AV to the sum of the unimodal activity (as the Reviewer proposes) yields the exact same differences as comparing AV – V to A (which is what we did).

Discussion:

One difference between fusion and combinations that is not discussed is that the total number of phonemes perceived is the definition of fusion. Fusion occurs in all congruent presentations and the "fusion"perceptions - except for the combination. It's not simply that the combination perception also includes an extra phoneme perceived, which is completely accurate, but it's also that fusion occurs in the congruent trial. The combination trial is fundamentally different from the congruent trials in a way that the fusion trial is not.

Response: This is a good point, which we now added on page 12.

Pg 10 – if the p2 is thought to represent feedback from STS, this should be discussed in the introduction.

Response: We have now included the rationale on page 4.

Pg 11 – "However, participants usually do not notice the AV incongruency in combination stimuli (or in fusion stimuli)." – was this measured?

Response: No, we have not measured this directly, and we have changed the sentence accordingly (see page 12).

Pg 11 – define CV – Is this a typo that's supposed to be "AV" or do you mean "consonant-vowel"?

Response: We intended to say "consonant-vowel", which we now clarify.

Pg 12 – The discussion of correlational results is rather lacking. Given the graphs of individual data points, I am convinced of the fusion correlation, but not of the combination correlation (see comment about Fig 3 below). Regardless of how these correlations hold up, there needs to be much more in the way of interpreting them for this presented result to be of meaning for the reader.

Response: In hindsight, we agree that the presentation and discussion of the correlation analyses were not very informative. Given the fair comment made by this Reviewer about the combination correlation

(see next comment), and the issue about statistical significance of the correlations (raised by Reviewer 3), we decided to delete the correlation analyses from the manuscript. Instead, we now clarified the theoretical frame-work (in response to the comments made by the Editor and all Reviewers) and included more (details regarding the) analyses, also in response to the Editor and all Reviewers.

Fig 3 – It seem that the correlation with combination responses is driven by a minority of participants (7) that reported this perception at any meaningful level.

Response: Yes, the Reviewer is right, and we acknowledge that this makes it rather difficult to draw any firm conclusions about this particular correlation (which is why we had not done so in the original manuscript). As mentioned in our response to this Reviewer's previous comment, we have now deleted the correlation analyses to avoid blurring our analyses/discussion with marginal results.

Typographic:

Pg 3, “Such fusions do not always occur: changing the modality” – should be a semicolon.

Response: Corrected.

Pg 5 – I think “FFMPEG” is written “FFmpeg”

Response: The Reviewer is correct and we have changed the text accordingly

Reply to Reviewer 3

Major

1. I was wondering whether something have gone wrong in the analysis of AgVb minus Vb trials. I have seen many AV minus V subtraction approaches in AV speech studies but have, thus far, never observed such a large difference between AV minus V vs. A alone as illustrated in Fig. 2, right panel, light gray trace. The trace for the AgVb minus Vb trials simply does not fit to the other six traces (of Auditory “b” and Auditory “g”, which should actually be labeled “combinations and “fusion”). This could also explain why there is such a highly significant difference between Auditory vs. AV congruent for fusion trials, whereas there is no such difference for combination trials. Overall, these results raise skepticism and I would like to encourage the authors to thoroughly double-check all their analysis scripts.

Response: We can assure the Reviewer that the subtractions were made correctly and that the V_b data subtracted from A_gV_b (the combination, light grey trace, right panel) is exactly the same data as subtracted from A_bV_b (the black trace in the left panel).

Often, the N1/P2 ERP plots are cut off somewhere in between 300 - 500 ms (see for example Besle et al., 2004, Frtusova et al., 2013, Ganesh et al., 2014, Klucharev et al., 2003; Stekelenburg & Vroomen, 2013, van Wassenhove et al., 2005). If we had done so, the difference between the combination wave and the other waves for auditory /g/ would have been outweighed by the resemblance. That is, < 400 ms, the combination difference wave looks as expected (it is similar to other auditory /g/ stimuli), and the deviation from the other five difference waves only starts after ~400 ms. We believe this is the consequence of the

actual processing differences between the combination vs all other stimuli, which we now clarify on pages 4 and 12.

The Reviewer also suggests us to re-label the Figure columns, but we believe using Fusions and Combinations would not do justice to the A and AV congruent conditions plotted in the same graphs. We did however add the labels Fusion and Combination in the Figure legend to clarify the issue.

The last part of the Reviewers comment is not clear to us, as we don't see the 'highly significant difference' vs. the 'no such difference' contrast the Reviewer mentions. In fact, the A vs. AV - V difference for congruent trials is significant for both "b" and "g". Although the pattern is indeed stronger for A_b vs. A_bV_b - V_b than for A_g vs. A_gV_g - V_g, the similarities between the significant effects in Figure 2b are more striking than the differences.

2. In order to draw the conclusion that there are differences between combination and fusion trials the authors should run an ANOVA showing an interaction (instead of comparing the outcome of two t-tests; see Nieuwenhuis et al. Nat. Neurosci., 2011, 1105-1107).

Response: The interaction effect that supports our conclusions requires data from all (or at least, all AV - V) conditions to be included in one ANOVA. Ideally, the data should therefore be averaged across the same time-window for all conditions (as we explained in the text, we could not reliably determine peak amplitudes for all participants). Since it is clear from Figure 2 that the ERPs and difference waves for the auditory /b/ conditions are quite different that in the auditory /g/ conditions (especially at the P2, which is critical), we had decided to restrict our analyses to the pair-wise tests. We intended to increase confidence in our findings by including 13500 tests with FDR correction for each comparison between conditions (rather than a priori selecting certain electrodes or time-intervals), but we fully understand the reviewer's concern and therefore now include ANOVAs as well.

However, the issue of selecting the 'correct' time-window that represents the peak data across all conditions still remains, so we averaged data in broad 100 ms time-windows that capture the N1 and P2 across all conditions (100-200 ms for the N1, and 200-300 ms for the P2). These data (from electrode Cz) were entered in 3 (Stimulus type; A, AV congruent, AV incongruent) * 2 (Auditory component; /b/ vs. /g/) ANOVA, which did not show the interaction for the N1, but did show the interaction for the P2. The same pattern was observed in 2 (Stimulus type; AV congruent vs. AV incongruent) * 2 (Auditory component /b/ vs. /g/) ANOVAs where the A-only data was omitted. These findings clearly align with the pair-wise comparisons in Figure 2b.

The changes in the text can be found on pages 7,8 and 9.

3. I was wondering whether the correlations would still be significant if the authors would have corrected for multiple testing.

Response: The FDR significance threshold for the weakest of the two correlations would be .0125 (i.e., 2/8*.05), whereas the actual p-value is .017. So no, both correlations would be non-significant. In light of this Reviewer’s comment and the comments made by Reviewer 2 regarding the correlations, we decided to delete the correlation analyses altogether.

4. Do the authors have an explanation why there was not a McGurk fusion effect in the ERPs (i.e. compared to congruent). Such effects have been consistently reported.

Response: There actually were differences between the fusion and congruent ERP difference waves. For example, averaged activity at Cz in a 190-220 ms window (the deflection between the N1 and P2, see Figure 2b), is different, $t(31) = 2.21$, $p = .034$, $d = .392$, as is the difference in a 360 - 440 ms window, $t(31) = 2.40$, $p = .023$, $d = .432$. However, these effects lose their statistical significance in the FDR corrected pair-wise comparisons approach we used. We have now clarified this on page 10.

Minor

1. Use the labels “fusion” and “combination” consistently in all figures.

Response: We have added the labels in all figures (or at least, in the Figure legends).

2. The Abbreviations “A” and “V” should be spelled-out at first use in the abstract.

Response: Corrected.

3. P. 6.: “…was randomly assigned to a finger.” Was this done across subjects? If so, this should be stated.

Response: Yes, this is now clarified.

4. P. 7: “The V and AV epochs contained 200 ms…”. It is unclear why the AV epochs were computed twice.

Response: AV epochs were not computed twice. V onset preceded A onset with 520 ms (see ‘Stimuli’ section). For V stimuli, there is no sound onset and epochs contained 200 ms of data before V onset. For A stimuli, there is no V onset and the epochs contained 720 ms of data before sound onset. Since AV stimuli have both V and A onsets, the epochs consisted of 200 ms of data before V onset, followed by 520 ms of data before A-onset, followed by 1000 ms of data after A onset. We now clarified this on page 7.

2nd Editorial Decision 20 September 2017

Dear Dr. Baart,

Your revised manuscript was re-evaluated by external reviewers as well as by ourselves and we are pleased to inform you that it will be accepted for publication in EJN after dealing with the few minor points raised by the reviewers and ourselves. The reviewers' comments are listed below and simply need clarification of the text. On my (PB) reading of the paper I find that I don't understand the title; could it be made more accessible to the general readership of EJN? The abstract also is a bit too specialized for the general readership and contains many undefined abbreviations. Could you make this a bit more accessible and include a concluding statement? Finally, we need better resolution figures.

If you are able to respond fully to the points raised, we shall be pleased to receive a revision of your paper within 30 days.

Thank you for submitting your work to EJN.

Kind regards,

Paul Bolam & John Foxe

EJN European Journal of Neuroscience FENS Federation of European Neuroscience Societies

co-Editors in Chief, EJN

Reviews:

Reviewer: 1 (Kaisa Tiippana, University of Helsinki, Finland)

Comments to the Author
The authors have addressed all my concerns. Just the theoretical part in the end of the Introduction could be explained better, along the lines they have done in their elaborate response to my previous comment 2 on the discussion. This is just a suggestion to be taken or ignored.

Reviewer: 3 (Daniel Senkowski, Charité- Universitätsmedizin Berlin, Germany)

Comments to the Author
The authors have done a good job in revising the manuscript. I am still surprised about the AgVb minus Vb trace, especially about the absence of any slow wave drift at longer latency. Nevertheless, I trust the authors when they assure that they have double checked their scripts. I only have some minor comments:

1) Abstract: It seems unusual to have parentheses around the last sentence of the abstract. I am also not sure that the reader will instantly understand what is meant by "number of perceived consonants" (after reviewing this paper I understand it but the first time reader of the abstract may not understand that this refers to "d" or "bg". I have the same issue with the last sentence of the paper and would like to encourage the authors to try to find a sentence that is intuitively easier to understand (maybe by describing it in two sentences).
2) p.3: Alsius et al., 20014 -> Alsius et al., 2014
3) p. 5, Procedure: ...and half were AV -> ... and half were bimodal
4) p.7, to clarify the new sentence I would suggest the following: The V as well as the AV epochs contained 200 ms before onset of the video. Auditory onset lagged video onset by 520ms. Accordingly, the A epochs contained 720 ms (i.e. 200 ms before onset of the video) before sound onset.
5) p. 7: Does the < 5 uV/200 ms refer to the SD? Please clarify.
6) p.13: The last sentences of the revised MS is a bit confusing. I am concerned that the reader will understand that the authors refer to the quality of the illusory percept when calling it "features". Please revise.

Reviewer: 2 (Ryan Stevenson, University of Western Ontario, Canada)

Comments to the Author
Thank you for addressing the presented concerns, and apologies for the error in missing the AV-V compared to A.

Authors' Response 27 September 2017

Reply to Editor

I find that I don't understand the title; could it be made more accessible to the general readership of EJN?

Response: We agree that the title may have been too specific, and we have changed it to " Electrophysiological evidence for differences between fusion and combination illusions in audiovisual speech perception ".

The abstract also is a bit too specialized for the general readership and contains many undefined abbreviations. Could you make this a bit more accessible and include a concluding statement?

Response: We have deleted the abbreviations from the abstract and included the following concluding statement: "... We argue that these effects arise because the phonetic incongruency is solved differently for both types of stimuli..."

Finally, we need better resolution figures.

Response: All figures are now uncompressed at a resolution of 800 dpi.

Reply to Reviewer 1

Minor

Just the theoretical part in the end of the Introduction could be explained better, along the lines they have done in their elaborate response to my previous comment 2 on the discussion. This is just a suggestion to be taken or ignored.

Response: We acknowledge that we do not provide a lot of information or elaboration, but we do believe all the essential information (including some key references) to follow our line of thought is already included in the paper on page 4:

" *Although phonetic AV integration is reflected at the P2 (Baart et al., 2014), the complete process requires a subsequent feedback loop that involves STS (Arnal et al., 2009). Therefore, we hypothesized that differences in AV integration patterns between McGurk fusions and combinations at/after the P2, could hint at differences related to congruency processing*..."

Reply to Reviewer 3

Minor

1) Abstract: It seems unusual to have parentheses around the last sentence of the abstract. I am also not sure that the reader will instantly understand what is meant by "number of perceived consonants" (after reviewing this paper I understand it but the first time reader of the abstract may not understand that this refers to "d" or "bg". I have the same issue with the last sentence of the paper and would like to encourage the authors to try to find a sentence that is intuitively easier to understand (maybe by describing it in two sentences).

Response: We agree that the essence of the 'number of perceived consonants' argument is hard to grasp from the abstract. We have therefore deleted it from the abstract, and we have also changed the final sentence of the manuscript to make it clearer.

2) p.3: Alsius et al., 20014 -> Alsius et al., 2014

Response: Corrected.

3) p. 5, Procedure: ...and half were AV -> ... and half were bimodal

Response: Corrected.

EJN European Journal of Neuroscience FENS Federation of European Neuroscience Societies

4) p.7, to clarify the new sentence I would suggest the following: The V as well as the AV epochs contained 200 ms before onset of the video. Auditory onset lagged video onset by 520ms. Accordingly, the A epochs contained 720 ms (i.e. 200 ms before onset of the video) before sound onset.

Response: Corrected.

5) p. 7: Does the < 5 uV/200 ms refer to the SD? Please clarify.

Response: No, the 0.5 µV is essentially a measure of inactivity: activity < .5 µV per 200 ms was considered an artifact, as mentioned in the text (it reads: "... *artifacts (amplitude changes ... or < .5 µV/200 ms)*..."). Since we believe the reviewers comment stems from accidentally misreading ".5 µV" as "5 µV", we have not changed the text.

6) p.13: The last sentences of the revised MS is a bit confusing. I am concerned that the reader will understand that the authors refer to the quality of the illusory percept when calling it "features". Please revise.

Response: We have revised the final sentence that no longer includes 'features'.
